# Empowering students to bridge basic and clinical sciences by creating innovative optometric tools

**DOI:** 10.1111/medu.15622

**Published:** 2025-02-19

**Authors:** Tsz Wing Leung

## 1 WHAT PROBLEMS WERE ADDRESSED?

Medical education often positions students as ‘users’ rather than ‘developers’ of tools and technologies. This reliance on external manufacturers can stifle innovation and limit the ability to create solutions tailored to specific patient needs. This challenge is particularly pronounced in optometry education, especially when teaching binocular vision therapy. Students must learn about various ocular motor control disorders and corresponding vision training strategies, but existing tools often have limitations and fail to address the diverse needs of patients, particularly children. To address this, we implemented an innovative teaching activity in which students became ‘developers’ to create novel binocular vision training tools. This approach reinforces fundamental basic science knowledge,^1^ such as understanding how the visual system integrates images from both eyes—crucial for developing effective therapies for conditions like lazy eye, squint and other ocular motor disorders. This project represents a new activity within our optometry program, effectively bridging the gap between basic science and clinical application.

## 2 WHAT WAS TRIED?

Forty six 4‐year optometry students (the year in our 5‐year program dedicated to clinical binocular vision) were tasked with developing effective and engaging binocular vision training tools. The project began with a collaboration with our University's Industrial Center, where students received an introduction to 3D printing and laser cutting. After gaining familiarity with these technologies, students reviewed existing binocular vision training tools and discussed their limitations. Throughout the semester, clinical faculty specializing in binocular vision therapy mentored students, providing guidance and feedback on their designs and prototypes. The semester culminated in a mini‐exhibition where students showcased their prototypes to clinical and academic staff. This event fostered significant student engagement, with students actively presenting and testing each other's creations. This approach, emphasizing the integration of basic science principles with clinical applications, created a highly interactive and practical learning experience.

## 3 WHAT LESSONS WERE LEARNED?

This project resulted in eight novel prototypes. One notable example is a 3D board game designed to improve binocular vision. The game utilizes red‐green anaglyph glasses. Against a white background, the eye looking through the red filter perceives only green targets, while the eye looking through the green filter perceives only red targets. This interactive game design compels patients to use both eyes, thus strengthening the ‘lazy eye’. While health care practitioners are not traditionally trained as developers, this pedagogical shift empowered students to think innovatively, consider patient needs and solidify their foundational knowledge.

Survey data revealed strong student agreement that the project enhanced their understanding of the link between basic and clinical sciences. Student feedback was overwhelmingly positive. One student commented, ‘The project on creating new vision therapy tools was incredibly helpful for my learning, as it required a deep understanding of the underlying principles’. Another student enthusiastically recommended, ‘Please continue this project next year! It's very meaningful and encourages creative thinking’. This educational strategy holds significant promise for adaptation across various medical disciplines, offering an interactive and effective method for reinforcing basic science knowledge and its practical application in clinical settings.

## AUTHOR CONTRIBUTIONS

Tsz Wing Leung was responsible for the conception, design and implementation of the project. He also led the data collection, data analysis and manuscript writing.

## CONFLICTS OF INTEREST STATEMENT

None.

## ETHICS STATEMENT

This project does not require ethical approval.

## Data Availability

The data that support the findings of this study are available from the corresponding author upon reasonable request.
